# Isolation and Characterisation of Human-Derived *bla*_KPC-3_-Producing *Salmonella enterica* Serovar Rissen in 2018

**DOI:** 10.3390/antibiotics12091377

**Published:** 2023-08-28

**Authors:** Daniela Fortini, Aurora García-Fernández, Claudia Lucarelli, Anna Maria Dionisi, Sergio Arena, Slawomir Owczarek, Michele Equestre, Alessandra Carattoli, Federica Sacco, Stefano Rossi, Roberta Ortenzi, Sara Primavilla, Laura Villa

**Affiliations:** 1 Department of Infectious Diseases, Istituto Superiore di Sanità, 00161 Rome, Italy; daniela.fortini@iss.it (D.F.); aurora.garciafernandez@iss.it (A.G.-F.); claudia.lucarelli@iss.it (C.L.); annamaria.dionisi@iss.it (A.M.D.); sergio.arena@iss.it (S.A.); slawomir.owczarek@iss.it (S.O.); 2 Department of Neurosciences, Istituto Superiore di Sanità, 00161 Rome, Italy; michele.equestre@iss.it; 3 Department of Molecular Medicine, Sapienza University of Rome, 00161 Rome, Italy; alessandra.carattoli@uniroma1.it (A.C.); federica.sacco@uniroma1.it (F.S.); 4 Hospital Castiglione del Lago, USL Umbria n.1, 06061 Perugia, Italy; stefano.rossi@uslumbria1.it; 5 Istituto Zooprofilattico Sperimentale dell’Umbria e delle Marche “T. Rosati”, 06126 Perugia, Italy; r.ortenzi@izsum.it (R.O.); s.primavilla@izsum.it (S.P.)

**Keywords:** whole-genome sequencing, *Salmonella* Rissen, antimicrobial resistance, *bla*_KPC-3_, pKpQIL plasmid

## Abstract

In this study, we describe a *Salmonella enterica* serovar (*S*.) Rissen strain with a reduced susceptibility to meropenem, isolated from a urinary infection in an 89-year-old woman in 2018 during activity surveillance in Italy (Enter-Net Italia). The genomic characteristics, pathogenicity, and antimicrobial resistance mechanisms were investigated via a genomic approach. Antimicrobial susceptibility testing revealed a “susceptible, increased exposure” phenotype to meropenem in the *S*. Rissen strain (4_29_19). Whole-genome sequencing (WGS) was performed using both the NovaSeq 6000 S4 PE150 XP platform (Illumina, San Diego, CA, USA) and MinION (Oxford Nanopore). The *S.* Rissen 4_29_19 strain harboured two plasmids: a pKpQIL-like plasmid carrying the *bla*_KPC-3_ resistance gene in a Tn*4401a* transposon (pKPC_4_29_19), and a ColE-like plasmid (p4_4_29_19) without resistance genes, highly prevalent among Enterobacterales. Comparative analysis revealed that the pKPC_4_29_19 plasmid was highly related to the pKpQIL reference plasmid (GU595196), with 57% coverage and 99.96% identity, but lacking a region of about 30 kb, involving the FIIK_2_ replicon region and the entire transfer locus, causing the loss of its ability to conjugate. To our knowledge, this is the first time that a pKpQIL-like plasmid, carrying *bla*_KPC-3_, highly diffused in *Klebsiella pneumoniae* strains, has been identified in a *Salmonella* strain in our country. The acquisition of *bla*_KPC_ genes by *Salmonella* spp. is extremely rare, and is reported only sporadically. In zoonotic bacteria isolated from humans, the presence of a carbapenem resistance gene carried by mobile genetic elements, usually described in healthcare-associated infection bacteria, represents an important concern for public health.

## 1. Introduction

The rapid spread of carbapenem-resistant Enterobacterales is emerging as a growing worldwide public health threat, mainly due to the production of carbapenemase enzymes causing limited therapeutic options to treat such infections [1]. Carbapenem resistance is often conveyed by carbapenem-resistant beta-lactamase (*bla*) genes found on mobile genetic elements (MGEs), such as integrons, insertion sequences, transposons, and plasmids. These MGEs can spread across bacterial cells of the same or different species. Carbapenem resistance emerged worldwide from the 2000s, mainly in *Klebsiella pneumoniae*, due to the diffusion of the *bla*_KPC_ gene, encoding the class A KPC enzymes, carried by high-risk clones of clonal group 258 (CG258, including sequence types ST258 and ST512) and ST307 [2,3,4]. The *bla*_KPC_ genes are generally located on Tn*4401*, a Tn*3*-like transposon [1,5,6]. It has been detected in the plasmids of several incompatibility groups, including IncFIIK plasmids related to pKpQIL [7], IncN, ColE [8,9], IncI2, and IncX3 plasmids [10,11]. This enzyme was initially described in *K. pneumoniae*, and is now readily detected in many members of the Enterobacterales family, but very rarely in *Salmonella enterica* (*S.*) [5,6,7,12,13]. The *bla*_KPC-3_ gene has been mainly described in pKpQIL plasmids in *K. pneumoniae* CG258 in Italy, and more recently, also in other *K. pneumoniae* lineages, including ST307. pKpQIL-like plasmids usually carry one or both the IncFIIk and FIBk replicons, and a cluster of approximately 35 kb encoding the conjugation machinery of pKpQIL [8,10,14,15]. The mating pair stabilisation mediated by TraN drives the high-efficiency transfer of IncF plasmids, supports the role of shaping the plasmid host range, and plays a prominent role in pKpQIL dissemination [16]. The dissemination of the pKpQIL plasmids may evolve in the rearrangement of the plasmid scaffolds with the loss of the *tra* region, the IncFIIk replicon, or resistance genes [17].

The minimum inhibitory concentration (MIC) to carbapenems may exhibit significant variation, depending on the membrane permeability status, the rate of carbapenem hydrolysis by the associated enzyme, and the gene expression level [18,19].

*Salmonella enterica* is one of the most important zoonotic foodborne pathogens causing human gastroenteritis. It comprises more than 2600 antigenically different serovars [20]. In the last decade, *S*. Rissen, an uncommonly reported serotype in humans, has been reported to play a significant role in the outbreak of foodborne diseases in Asian countries [21]. The role of this serotype has also been highlighted in Europe, and it is frequently reported in the carcasses of food-producing animals, contributing to the spread of resistance in *Salmonella* spp. On the other hand, cases of *S.* Rissen in humans are not abundant [22].

This study describes the phenotypic and genotypic analysis of a *bla*_KPC_-producing *S*. Rissen strain (4_29_19) isolated in Italy in 2018, during activity surveillance (Enter-Net Italia) from a woman’s urine sample. This strain showing a reduced susceptibility or a susceptible, increased exposure to meropenem. A susceptible, increased exposure has been defined by EUCAST when there is a high likelihood of therapeutic success, because exposure to the agent is increased through adjustment of the dosing regimen, or by its concentration at the site of infection (https://www.eucast.org/newsiandr, accessed on 10 December 2022). *Salmonella* urinary tract infections are uncommon in routine hospital practice, and are infrequently documented in the scientific literature, although they may cause serious morbidity in immunocompromised, and even in immunocompetent, patients [23]. The presence of certain pathogenesis islands (SPIs) or single virulence genes could represent an advantage for bacteria to establish the infection. The whole genome sequencing (WGS) approach elucidates the antimicrobial resistance (AMR) mechanisms, plasmid evolution, and chromosomal characteristics of this isolate.

## 2. Results

*S*. Rissen 4_29_19 was resistant to tetracycline, amikacin, and almost all beta-lactam antibiotics, including ertapenem and imipenem carbapenems, and showed a “susceptible, increased exposure” phenotype to meropenem, with an inhibition zone diameter of 18 mm and MIC = 4 mg/L (Table 1). The strain was susceptible to the ceftazidime/avibactam combination, tigecycline, colistin, ciprofloxacin, fosfomycin, gentamicin, trimethoprim/sulfamethoxazole, and nitrofurantoin. The KPC enzyme was detected in the 4_29_19 strain, via an immunochromatographic assay (Supplementary Figure S1).

The FullForcePlasmidAssembler (FFPA.py), performing an Illumina and Nanopore WGS hybrid assembly, generated a circularised chromosome and two circularised plasmid sequences.

The chromosome was 4,919,121 bp in size, and the GC content was 52.1%, containing 4538 coding sequences (CDSs), 22 rRNAs, and 86 tRNAs. The multilocus sequence typing (MLST) obtained through the chromosomal sequence resulted in ST469. The in silico analysis for the presence of antimicrobial resistance genes in the chromosome identified *tet*(A) and *aac*(6′)*-Iaa* genes; the last one is intrinsic to the *Salmonella* genus, and it is considered a cryptic gene that does not contribute to aminoglycoside resistance [24]. According to SPIFinder, 4_29_19 strain contained the following pathogenicity SPIs: SPI-1 to SPI-5, SPI-8, SPI-9, and C63PI. The numerous gene clusters located in SPIs are implicated in the pathogenesis of *Salmonella* spp. The five SPI-1-5 are common to all serotypes of *Salmonella* spp. SPI-1, SPI-2, SP4, SP8, and SPA9 modulate bacterial invasion into intestinal epithelial cells; SPI-3 is involved in the survival of *Salmonella* spp. in macrophages (intracellular survival and replication). SPI-5 encodes five genes (*pipA*, *pipB*, *pipC*, *pipD*, and *sopB*) involved in intestinal mucosal fluid secretion and inflammatory responses, and C63PI encodes an iron transport system [25].

The *Salmonella* 4_29_19 strain presented several virulence genes, on the chromosome, linked with the pathogenesis of *Salmonella* spp. [26], involving adhesion systems, iron uptake, magnesium uptake and manganese uptake, motility, and type III secretion systems (T3SS) (Table 2).

**Figure 1:**
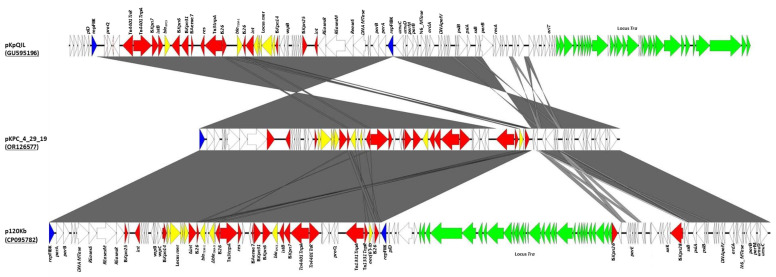
Comparison of the plasmid sequences of pKPC_4_29_19 (OR126577), pKpQIL (GU595196), and p120Kb (CP095782), using the Easyfig program. Yellow arrows indicate the resistance genes; blue arrows indicate the replication genes; red arrows indicate the mobile genetic elements; green arrows indicate the tra locus; and white arrows indicate the genes involved in plasmid duplication and stability, and hypothetical proteins.

The *Salmonella* 4_29_19 strain harboured two plasmids: an IncFIB (pKpQIL)-like plasmid of 70,085 bp (pKPC_4_29_19), carrying various resistance genes, and a ColE-like plasmid of 4657 bp (p4_4_29_19), without antibiotic resistance genes, highly prevalent among Enterobacterales. The pKPC_4_29_19 plasmid harboured *bla*_TEM-1_ and a truncated *bla*_OXA-9_ gene between two IS*26* elements; a *bla*_KPC-3_ gene located in Tn*4401a*, a Tn*3*-like element; an *aac(6')-Ib* gene positioned 15,260 bp downstream of the *bla*_KPC-3_ gene and flanked by a Tn*3* transposon and an IS*26*; and a *mer* operon, conferring resistance to mercuric ions (Figure 1). The pKPC_4_29_19 plasmid carried the toxin/antitoxin *vapB*/*vapC* systems and *parB/parM* partitioning genes encoding proteins involved in plasmid stability. Comparative analysis of the pKPC_4_29_19 plasmid was performed using the reference pKpQIL plasmid (GU595196), the *bla*_KPC-3_-carrying plasmid harboured by the carbapenem-resistant *K. pneumoniae* clone ST258 isolated in Israel [14], and the p120Kb (CP095782) plasmid, showing the best match using BLASTN, isolated in Italy from a *K. pneumoniae* strain co-producing OXA-181 and KPC-121, a KPC variant conferring resistance to ceftazidime/avibactam (Figure 1) [27]. The plasmid comparison revealed that the reference pKpQIL plasmid (GU595196) [14] showed 57% coverage, and 99.96% nucleotide identity, with pKPC_4_29_19. Similarly, the p120Kb (CP095782) plasmid [27] displays 58% coverage and 99.99% identity with the pKPC_4_29_19 plasmid. With respect to the reference pKpQIL plasmid (GU595196), the pKPC_4_29_19 plasmid showed a deletion of 49,520 bp, including the FIIK_2_ replicon and the entire transfer locus regions. The deletion of the transfer locus caused the loss of self-conjugation of pKPC_4_29_19 (Figure 1). Conjugation experiments on the pKPC_4_29_19 plasmid were not successful. This deletion was probably mediated by the MGE regions. It is plausible that significant rearrangements of the plasmid scaffold could be caused by the presence of six copies of IS facilitating homologous recombination through the exchange of sequences between identical or related segments.

Interestingly, the pKPC_4_29_19 plasmid presented a region of 5969 bp, encoding the Tn*1331* transposon, *aac(6')-Ib*, and IS*26*, which has been very rarely reported in pKpQIL-like plasmids (identified in p120Kb and pKPN39428, CP056026) (Figure 1). The Tn*1331* was identified to be associated with the *bla*_KPC-3_ gene in *K. pneumoniae* strains isolated in Jerusalem from 2005 to 2009, but it was located on a different plasmid type, and not on pKpQIL [28].

**Figure 2:**
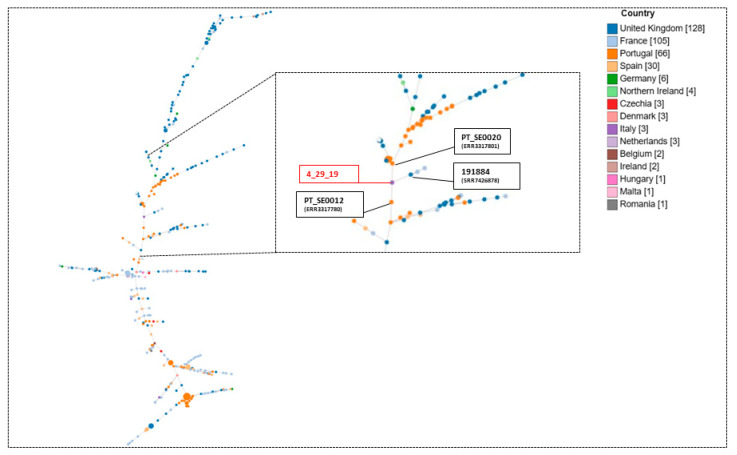
The phylogenetic relationship between the *S.* Rissen strain 4_29_19 and other *S.* Rissen strains belonging to ST469 available in the EnteroBase database in February 2023 (strains with differences of less than 500 AD from cgMLST) via cgMLST analysis. The colours indicate different countries.

To investigate the genomic characteristics of the *S.* Rissen strain in a global context, the phylogenetic analysis relationship between *S*. Rissen 4_29_19 and 359 *S.* Rissen isolates belonging to ST469 in Europe (retrieved from EnteroBase in February 2023) was performed. The core genome Multilocus Sequence typing (cgMLST) analysis performed via the chewBBACA (BSR-Based Allele Calling Algorithm) revealed that the *S.* Rissen isolates were widely distributed in Europe, differing by 1 allele distance (AD) from 81 AD. Italian isolates were poorly represented (three isolates), and scattered along the tree, making it impossible to identify a geographical cluster (Figure 2). The closest relatives of *S*. Rissen 4_29_19 were the isolates PT_SE0020 (ERR3317801) and PT_SE0012 (ERR3317780) recovered in 2015 in humans and pigs in Portugal, differing by 40AD and 34AD, respectively, and the 191,884 (SRR7426878) strain, isolated in the UK in humans, differing by 34 AD. PlasmidFinder analysis revealed that these isolates did not carry plasmids.

## 3. Discussion

*S.* Rissen in the European Union (EU) is among the top 20 serovars recovered from humans and animals (pigs and bovines) in 2021 [29]. Different virulence genes are involved in the pathogenicity of *Salmonella* spp., which contributes to their invasion and proliferation in complex environments. The virulence components, including adhesins, invasins, and toxins, frequently cluster in specific chromosomal regions, known as SPIs. *S*. Rissen is characterised by a variety of genetic patterns accounting for its virulence and antibiotic resistance [30]. The *S*. Rissen 4_29_19 strain contains important SPIs and many virulence-associated genes, which highlight its pathogenesis. In recent years, an increase in multi-drug resistant (MDR) *S.* Rissen isolates has been described. Most of the *S.* Rissen individuals isolated from pigs were MDR (93%). The most diffused resistance pattern is ampicillin, chloramphenicol, sulfamethoxazole, tetracycline, and trimethoprim, mainly carried on integrons [31,32]. In some parts of Asia, *S.* Rissen is also a common serovar in pigs, chickens, pork, and humans [33]. It has been demonstrated that *S*. Rissen isolates from Thai pig farms were frequently MDR to most of the antimicrobials listed above. Considering the risk associated with *Salmonella* spp. contamination, humans can acquire bacterial infections containing antimicrobial-resistant genes through the consuming or handling of contaminated food [29,34,35]. No meropenem-resistant human isolates were reported in the EU from 2020 to 2021, while one human isolate was identified in 2019 (OXA-48 producing *S*. Typhimurium var. Copenhagen, isolated from a domestically acquired infection in Spain) [36,37]. In 2018 and 2021, none of the *Salmonella* spp. isolates recovered from animals or carcasses were reported as being resistant to meropenem [34]. However, it should be noted that, in some European reporting countries, the meropenem results were interpreted using the European Committee on Antimicrobial Susceptibility Testing (EUCAST) clinical breakpoint, which is four dilutions higher than the ECOFF, leading to a sub-detection of isolates with carbapenem resistance genes, not conferring full resistance. EUCAST suggests, for carbapenemase, a meropenem screening cut-off of >0.125 mg/L (zone diameter < 28 mm) (http://www.eucast.org, accessed on 10 December 2022).

We reported the description of an *S*. Rissen isolate with resistance to imipenem and ertapenem, and a reduced susceptibility to meropenem, that produced the KPC-3 enzyme. In Italy, the most frequent carbapenemase is KPC-3. In the last decade, KPC-3-producing *K. pneumoniae*, belonging to CG258, has been responsible for a high incidence of invasive, nosocomial-acquired infections in our country [15,38]. The resistance to carbapenems is mainly based on the horizontal gene transfer of plasmids carrying multiple resistance genes [1], and the most frequent plasmid to be associated with the *bla*_KPC-3_ gene is the pKpQIL-like plasmid. Several reports have already described the spread of this plasmid type among different bacterial isolates and patients during hospital outbreaks [39]. The description of the plasmid provides interesting confirmatory insights into the plasticity of pKpQIL-like plasmids, and their role in the ability to adapt to different bacterial species and genera by facilitating the spread of *bla*_KPC-3_. These plasmids were isolated in clinically relevant Enterobacterales across the globe [11,40], but pKpQIL complete sequences are present in the NCBI database only in isolates of *Klebsiella* spp., *E. coli*, *Enterobacter cloacae*, and *Raoultella ornithinolytica*. The presence of *bla*_KPC-3_ in a pKpQIL-like plasmid is described in *Salmonella* spp. for the first time in this study. The acquisition of a KPC-3 positive pKpQIL plasmid by *S.* Rissen could be explained by the clinical history of the patient from whom the *S*. Rissen strain 4_29_19 was isolated. That patient, indeed, during previous hospitalisations, presented an acute urinary infection caused by ESBL-producing *Klebsiella* spp., which could be responsible for the diffusion of the pKpQIL-like plasmid to the *S*. Rissen strain.

As previously described, our results indicated that pKpQIL-like plasmids are in constant genetic flux, creating new pKpQIL plasmid variants [7,9]. The reported excision of the replicon region FIIK_2_ and the entire transfer locus in pKPC_4_29_19 is a non-rare event. The plasticity of pKpQIL plasmids due to genetic rearrangements has been highlighted several times. In the pKpQIL-SC29 plasmid (JX442977), a reversion of the strain to carbapenem susceptibility was probably caused by IS*26*-mediated looping out, resulting in the deletion of the entire Tn*4401*-*bla*_KPC-3_ transposon and transfer locus in the plasmid [17]. The expression of *bla*_KPC-3_ in *S.* Rissen conferred resistance to imipenem and ertapenem carbapenems, and increased the MICs for meropenem (from MIC < 0.12 mg/L in a control strain to MIC = 4.0 mg/L in *S*. Rissen strain 4_29_19), a phenotype compatible with an efficient membrane permeability, as described in other species [13]. The acquisition of *bla*_KPC_ genes by *Salmonella* spp. is highly unusual. It has been reported only sporadically in the *S.* Javiana strain from Brazil, where *bla*_KPC-2_ was carried by a small IncQ1 plasmid, or in *S.* Schwarzengrund in Argentina, where *bla*_KPC-2_ was present in an IncL/M plasmid, or in *S.* Typhimurium in China, where *bla*_KPC-2_ was located in an IncX4 plasmid [12,13,41]. Other authors reported the presence of *bla*_KPC-2_ in the serovars Cubana, Javiana, and Typhimurium, from the United States, Paraguay, and Colombia, respectively, but lacked, however, genotyping studies [12,13,42,43,44]. Interestingly, the serovars Schwarzengrund and Javiana displayed a similar low-level resistance to meropenem to that of the *S*. Rissen 4_29_19 isolate [12,13].

The rare conjugative acquisition of pKpQIL by *Salmonella* spp. isolates is probably due to the lack of a wild-type OmpK36 porin in this species. It has been demonstrated that the mating pair stabilisation during the conjugation process requires the interaction between the TraN factor of pKpQIL and OmpK36 present in *K. pneumoniae*, but absent in *Salmonella* spp. [16]. It could be hypothesised that the acquisition of the pKpQIL plasmid by the *S*. Rissen strain 4_29_19 occurred due to an event of conjugation supported in trans by a co-resident plasmid. 

## 4. Materials and Methods

### 4.1. Phenotypic Analysis

The *S.* Rissen 4_29_19 strain was tested for antimicrobial susceptibility via the disk diffusion method and broth microdilution method, following the EUCAST guidelines (https://www.eucast.org/fileadmin/src/media/PDFs/EUCAST_files/Breakpoint_tables/v_13.1_Breakpoint_Tables.pdf, accessed on 10 December 2022). A susceptibility test was performed using a 17-antimicrobial panel (Liofilchem S.r.l., Roseto degli Abruzzi (TE), Italy). The antibiotics tested via the disk diffusion method were: nalidixic acid (NA, 30 µg), pefloxacin (PEF, 5 µg), ampicillin (A, 10 µg), cefotaxime (CTX, 5 µg), ceftazidime (CAZ, 10 µg), cefoxitin (FOX, 30 µg), amoxicillin/clavulanic acid (AMC, 20 µg/10 µg), meropenem (MEM, 10 µg), chloramphenicol (C, 30 µg), gentamicin (G, 10 µg), kanamycin (K, 30 µg), streptomycin (S, 10 µg), sulphamethoxazole (Su, 0.25 µg), kanamycin (K, 30 µg), tetracycline (T, 30 µg), trimethoprim (TMP, 5 µg), and trimethoprim/sulphamethoxazole (SXT, 1.25 µg/23.75 µg). 

The MIC of antibiotics tested was determined using the MicroScan WalkAway system (Beckman Coulter, Inc., Brea, CA, USA). 

The *Salmonella* 4_29_19 strain was tested via an immunochromatographic assay for the detection of the five most common carbapenemase producers (KPC, IMP, NDM, VIM, and OXA-48) (NG-CARBA 5; Biotech, Guipry, France). 

The conjugation experiments of the pKPC_4_29_19 plasmid were performed using rifampicin-resistant *E. coli* DH5-α and Luria–Bertani agar plates (A, 10 mg/L and rifampicin 50 mg/L) at 37 °C. 

### 4.2. Genomic Analysis

The genomic DNA was purified using the ISOLATE II Genomic DNA Kit (Bioline, Memphis, Tennessee 38134-5611 USA). WGS was performed with the NovaSeq 6000 S4 PE150 XP platform, using a standard genomic library (Illumina, San Diego, CA, USA), and via MinION Mk1C, using a Rapid Barcoding Kit (SQK-RBK004) and R9.4 FLO-MIN106 Flow Cell (Oxford Nanopore Technologies). The integrated analysis of the genomic sequences obtained using the NovaSeq 6000 Illumina and MinION platforms was performed using the FullForcePlasmidAssembler (FFPA.py) pipeline (https://github.com/MBHallgren/FullForcePlasmidAssembler, accessed on 20 January 2023). FullForcePlasmidAssembler uses Trimmomatic and QCAT for trimming Illumina and Nanopore data, respectively. FastQC and Nanoplots are used to quality-check the trimmed reads. Kraken reports are generated from the reads, and then Unicycler is used to perform a hybrid assembly. Finally, Abricate via plasmidfinder_db and resfinder_db is run (https://onehealthejp.eu/projects/antimicrobial-resistance/jrp-full-force, accessed on 20 February 2023). Plasmid sequence annotation was performed with a Bakta pipeline (https://www.uni-giessen.de/de/fbz/fb08/Inst/bioinformatik/software/bakta, accessed on 15 February 2023), and the ISs were manually curated using ISfinder (https://isfinder.biotoul.fr/, accessed on 15 February 2023). The plasmid and resistance gene content was obtained using the PlasmidFinder and ResFinder tools (https://cge.cbs.dtu.dk/services/, accessed on 15 February 2023), respectively. The *Salmonella* spp. sequence typing was performed using the *Salmonella* typing database MLST v2.0 tool (https://pubmlst.org/bigsdb?db=pubmlst_salmonella_seqdef, accessed on 15 February 2023). The phylogenetical analysis was performed using a core genome multilocus sequence typing (cgMLST) strategy, using the chewBBACA BSR-Based Allele Calling Algorithm (Galaxy Version 2.0). The 359 European isolates of *S.* Rissen ST469, from 15 different countries, were retrieved from EnteroBase in February 2023, and were used for this analysis. The cgMLST phylogenetic tree was constructed and visualised via the FigTree v1.4.4 software. The pathogenicity genetic repertoire of the *S*. Rissen isolate, and its virulence factors were detected using SPIFinder 2.0 (https://cge.food.dtu.dk/services/SPIFinder, accessed on 15 February 2023). 

The sequence comparison with the closely related plasmid (CP095782) and the reference pKpQIL plasmid (GU595196) was performed using BLAST, and visualised via Easyfig v 2.2.3 (https://mjsull.github.io/Easyfig/, accessed on 20 February 2023).

## 5. Conclusions

The identification, in zoonotic bacteria isolated from humans, of carbapenem resistance genes carried by MGE, usually described in healthcare-associated infections, is an important concern for public health. Our findings reinforce the importance of maintaining active AMR surveillance in *Salmonella* spp. responsible for community infections. Integrated AMR surveillance in nosocomial and veterinary infections must be promoted and consolidated. 

## Figures and Tables

**Table 1:** The MIC in mg/L measured for the *Salmonella enterica* Rissen 4_29_19 strain using the MicroScan WalkAway system (Beckman Coulter, Inc., Brea, CA, USA).

Antibiotic	MIC Value	MIC Interpretation *
Amikacin	>16	R
Ampicillin	>8	R
Amoxicillin–clavulanic acid	>32	R
Ceftazidime	>32	R
Cefotaxime	>32	R
Cefoxitin	16	R
Colistin	≤2	S
Ciprofloxacin	≤0.06	S
Cefepime	>8	R
Ceftazidime/Avibactam	≤2	S
Nitrofurantoin	≤64	S
Fosfomycin	≤16	S
Gentamicin	≤2	S
Imipenem	8	R
Meropenem	4	I
Ertapenem	>1	R
Trimethoprim/sulfamethoxazole	≤2/38	S
Tigecycline	≤1	S

* In the MIC interpretation, R indicates resistant; I indicates susceptible, increased exposure; and S indicates susceptible. Standard dosing regimen, interpreted as indicated in the European Committee in their Antimicrobial Susceptibility Testing Breakpoint tables for the interpretation of MICs and zone diameters Version 13.1, valid from 2023-06-29.

**Table 2:** *Salmonella enterica* Rissen 4_29_19 virulence factors present in the chromosome.

Virulence Factors Classes	Virulence Factors	Genes
Fimbrial adherence determinants	Agf (thin aggregative fimbriae/curli)	*csgABCDEFG*; *steAC*
	Lpf (long polar fimbriae)	*lpfABCDE*
	Type 1 fimbriae	*fimCDFHI*
Non-fimbrial adherence determinants	SinH	*sinH*
	MisL	*misL*
Iron uptake	Enterobactin	*entCDEFHS*; *fepABCDG*
Magnesium uptake	Magnesium uptake/transporter	*mgtABCLRS*
Iron and manganese transport	Periplasmic-binding protein	*sitABCD*
Macrophage inducible gene	Antimicrobial peptide resistance protein Mig-14	*mig14*
Motility	Flagella	*cheABYWRVZ*; *flgABCDEFGHIJKL*
Secretion system	T3SS (SPI-1 encoded)	*invACEFGHR*; *orgABC*; *prgHIJK*; *sicAP*; *sipABCD*
	T3SS-1 translocated effectors	*avrA*; *sspABH2*; *sopABDF*
	T3SS (SPI-2 encoded)	*ssaUTSRQPONVMLKJIGDE*; *sseABCDE*; *sscAB*
	T3SS-2 translocated effectors	*pipABB2*; *sifAB*; *sopD2E2*; *sseFGJ*
Serum resistance	OmpA (outer membrane protein A)	*ompA*
Others	Lipooligosaccharide	*gmhB*/*lpcA*

## Data Availability

The genomic and plasmidic sequences have been deposited in GenBank under BioProject accession number PRJNA972423. The strains have been stored under the BioSample accession number SAMN34994532. The chromosome sequences were released under accession number CP126310. The manually curated plasmid sequences were released under the accession numbers OR126577 (pKPC_4_29_19) and OR126578 (p4_4_29_19).

## Supplementary Materials

The following supporting information can be downloaded at: https://www.mdpi.com/article/10.3390/antibiotics12091377/s1, Figure S1: The KPC enzyme was detected in the 4_29_19 strain, via an immunochromatographic assay.
