# Supplementary figures and images for: Isolation and Characterisation of Human-Derived blaKPC-3-Producing Salmonella enterica Serovar Rissen in 2018
Source: Antibiotics (Basel). 2023 Aug 28;12(9):1377. doi: 10.3390/antibiotics12091377 (PMC10525129; doi:10.3390/antibiotics12091377)

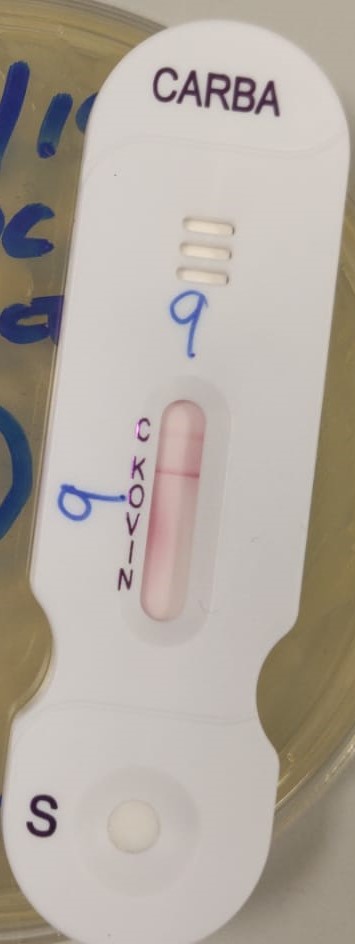

Supplement: Supplementary file 1 [file antibiotics-12-01377-s001.zip › antibiotics-2542661-supplementary.jpg]
